# Surgical treatment of gingival overgrowth with 10 years of follow-up

**DOI:** 10.1186/1746-160X-6-19

**Published:** 2010-08-12

**Authors:** Andrea Ballini, Adele Scattarella, Vito Crincoli, Roberto Gianfranco Carlaio, Francesco Papa, Letizia Perillo, Teodoro Romanazzo, Maria Virginia Bux, Gianna Maria Nardi, Angela Dituri, Stefania Cantore, Francesco Pettini, Felice Roberto Grassi

**Affiliations:** 1 Department of Dental Sciences and Surgery, University of Bari, Bari, Italy; 2 Department of Dental Sciences, University of Rome, "Sapienza", Rome, Italy; 3 Department of Orthodontics, University of Naples (Second University), Naples, Italy

## Abstract

**Background:**

In some pathological conditions, gingivitis caused by plaque accumulation can be more severe, with the result of an overgrowth. Nevertheless, the overgrowth involves the gingival margin with extension to the inter-dental papilla. The lesion may involve the inter-proximal spaces, and become so extensive that the teeth are displaced and their crowns covered. Severe overgrowth may lead to impairment in aesthetic and masticatory functions, requiring surgical excision of the excessive tissue. Aim of this study is to describe an operative protocol for the surgical treatment of localized gingival overgrowth analyzing the surgical technique, times and follow-up.

**Methods:**

A total of 20 patients were enrolled and underwent initial, non surgical, periodontal treatment and training sessions on home oral hygiene training. The treatment plan involved radical exeresis of the mass followed by positioning of an autograft of connective tissue and keratinized gingiva.

**Results:**

During 10 years of follow-up, all the grafts appeared well vascularized, aesthetically satisfactory, and without relapse.

**Conclusions:**

Periodontal examinations, surgical procedures, and dental hygiene with follow-up are an essential part of the treatment protocol. However, additional effort is needed from the patient. Hopefully, the final treatment result makes it all worthwhile.

## Background

The term gingival overgrowth (GO) only provides a topographic description of the lesion but no histological diagnosis.

Moreover, the histological classification is still unclear, owing to the wide range of possible histological morphotypes [1,2].

In fact, elements of granulation tissue are frequently observed, as are giant cells, mesenchymal cells combined or not with fibroblasts, collagen, epithelial cells, calcification zones and vessels [2].

From the epidemiologic point of view, GO most often affects the female sex, at ages ranging from 6 to 80 years but with a prevalence between the second and fifth decades of life [3,4].

The etiology is still unknown, although there is a consensus from some Authors that chronic local trauma (plaque, poor oral hygiene, defective restoration, foreign bodies such as food impaction or toothbrush bristle) can trigger chronic inflammation of the periodontal tissue, together with an endocrine or metabolic imbalance, which may determine the onset of the lesions [1,3,4].

Among the important systemic conditions in the etiopathogenesis of GO, hormonal factors must be borne in mind, which have a fundamental role in amplifying the tissue reaction to chronic inflammatory conditions [5].

In current clinical descriptive terminology, GO can be classificated as [1,3,6]:

A-) According to etiologic factors an pathologic changes, GO could be listed out as:

I-) Inflammatory overgrowth

a. Chronic

b. Acute

II-) Drug-induced overgrowth

III-) Overgrowth associated with systemic disease

a. Conditioned overgrowth

1. Pregnancy

2. Puberty

3. Vitaminic C deficiency

4. Plasma Cell gingivitis

5. Non- specific conditioned overgrowth (granuloma pyogenicum)

b. Systemic diseases causing gingival overgrowth:

1. Leukemia

2. Granulomatous diseases

IV-) neoplastic overgrowth (gingival tumors)

V-) False overgrowth

B-) According to location and distribution, gingival overgrowth can be classified as:

Localized: gingival overgrowth limited to one or more group of teeth

Generalized: Entire mouth

Papillary: Confined to the interdental papilla

Diffuse: Involves all the parts of the gingival, i.e. marginal, attached and interdental gingival

Discrete: isolated sessile or peduncolated tumor-like overgrowth.

Three different types of drugs are associated with GO, namely anti-convulsant, calcium channel blockers and the immunosuppressants like cyclosporine [6].

Cyclosporine A (CsA) has been the primary tool to prevent the rejection of organ transplants. CsA is still the mostly used drug in renal transplant therapy [6].

However, there is evidence that use of Tacrolimus causes fewer oral side-effects than CsA [7,8].

The histopathological classification of GO is as follows: gigant cell, fibromatous, peripheral ossification and congenital [1,2,9].

There are various, controversial theories as to the origin of those cells, whereby some Authors believe that they could derive from the osteoclasts, other Authors attribute them a mesenchymal origin, or an endothelial origin and yet other Authors consider that they derive from pericapillary adventitial cells [9-11].

Finally, the epithelial lining of the giant cell form is of multilayered type with signs of hyper- and para-keratosis combined with ulcerative phenomena [4].

The peripheral ossification form shows a histological drawn of layers of connective tissue with an irregular appearance and a rich content of bone trabeculae and calcified matter in the stroma [9,10].

Instead, in the third form of GO mature connective tissue is present, lined by a hyper-para-keratosic epithelium.

There is often a modest degree of aspecific inflammatory infiltrate[1-3].

In the past, treatment was obtained by complete exeresis of the mass and removal of the adjacent tooth or teeth to avoid recurrence, thus resulting in a very poor aesthetic and functional outcome [11].

Nowadays, classic treatment of GO is by surgical excision of the lesion with curettage of the dental and periodontal structures in the involved area, and histological analysis of the removed tissue [5,11,12].

Instead, some studies have proposed the use of laser treatment as a valid alternative to conventional surgical treatment [12-16].

According to these studies, traditional surgical excision is not only extremely difficult but also causes post-surgical pain, gingival deformity and a difficult post-surgical course.

All this can complicate home oral hygiene procedures and allow bacterial colonization, that can often delay patients healing [17,18].

Aim of this study was to describe an operative protocol for localized GO (using free soft tissue grafts), the surgical timing and follow-up.

In fact, as described before, a number of surgical procedures have been proposed to treat GO.

In this study it is used free soft tissue grafts, because this procedure increase the width of keratinised tissue and improve aesthetics results.

## Patients and Methods

### Case series presentation

We report on 20 patients (8 males and 12 females) with a mean age of 29 ± 4 months, with different etiopathogenesis of localized GO present from 15 days to 12 month (Table 1).

**Table 1:** Time of beginning, etiologic factor and associated disease distribution.

PATIENTS	AGE	SEX	DAYS/MONTHS SINCE ONSET	ETIOLOGIC FACTORS	OTHER DISEASES AND DRUGS THERAPY
1	25	M	15 days	Calculus	None

2	43	F	10 months	Unsuitable prosthesis	Diabetes mellitus (insulin)

3	25	F	5 months	Calculus	Psoriasis (betamethasone)

4	26	F	1 year	Calculus,	None (oral contraceptives)

5	35	M	7 months	Food Impaction	Sjogren's Syndrome (pilocarpine, carboxymethyl cellulose collirium)

6	42	M	10 months	Calculus	Epilepsy (phenylhydantoin)

7	68	F	1 year	Plaque, calculus	Hypertension (nifedipine)

8	50	F	8 months	Plaque, calculus	None

9	21	F	2 months	Plaque	None

10	33	M	2 months	Plaque	None

11	54	M	6 months	Unsuitable prosthesis	Rheumatoid arthritis

12	34	F	9 months	Calculus	Systemic lupus erythematosus (betamethasone)

13	65	M	1 year	Unsuitable prosthesis	osteoarthrosis

14	24	F	4 months	Calculus	None

15	39	F	1 year	Calculus	None (oral contraceptives)

16	43	F	2 years	Calculus, deciduous roots	Crohn's disease (prednisolone, azathioprine)

17	52	M	8 months	Unsuitable prosthesis	None

18	63	M	1 year	Calculus	Behcet 's disease (prednisolone)

19	56	F	10 months	Food Impaction	Osteoarthrosis

20	34	F	6 months	Calculus	None

Only two patients were in therapy with drugs that can influence GO (phenylhydantoin, nifedipine).

Also in those cases of drug-induced hyperplasia the GO were localized.

The study was performed in accordance with the Declaration of Helsinki [19] and the guideline for Good Clinical Practice [20].

All patients were able to give consent to participation in the study after receiving oral and written information.

Each patient underwent an initial non surgical periodontal treatment, with full-mouth tooth polishing and oral hygiene home instructions.

Home oral hygiene also included the use of a single tufted brush for the less accessible zones.

Patients were instructed to use a liquid plaque indicator (GC Plaque Indicator Kit^®^), to remove all visible plaque very meticulously with toothbrush and using a 1% chlorhexidine gel (Corsodyl dental gel^®^-GlaxoSmithKline -Brantford, Middlesex, UK).

Root debridement was carried out with manual and ultrasonic instruments to complete the baseline therapy.

This protocol was able to eliminate the local aggravation factors and thus guarantee a good surgical result.

**Figure 1:**
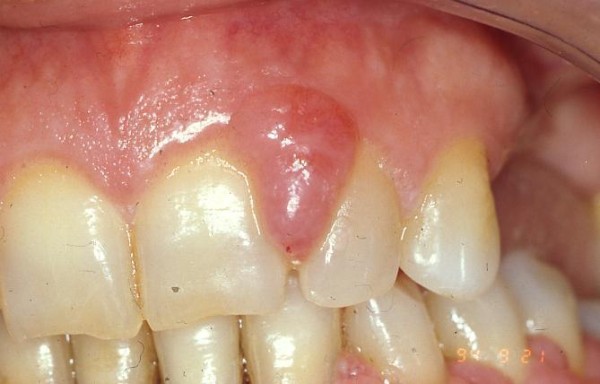
**Intraoral view of gingival overgrowth**.

In 12 patients the GO was localized in the upper jaw, between the central and the lateral (fig. 1) incisor (7 subjects), between the lateral incisor and the canine (5 subjects), while in the remaining 8 it was localized in the mandible between the canines.

Before non-surgical therapy, three different indexes of periodontal health were analyzed (for full mouth): probing depth (PD), plaque index according to 0'Leary (PI) [21] and Gingival index according to Löe-Silness (GI) [22,23]. (Table 2)

**Table 2:** Initial and final distribution of probe depth (P.D.), plaque index (P.I.) and gingival index (G.I.), before (T_0 _) and after (T_1_) non surgical therapy in 20 Patients (Pt) with different type of Epulides.

**Pt**.	**P.D**. **(T**_**0 **_**)**	**P.D**. **(T**_**1**_**)**	**P.I**. **(T**_**0 **_**)**	**P.I**. **(T**_**1 **_**)**	**G.I**. **(T**_**0 **_**)**	**G.I**. **(T**_**1 **_**)**	Epulides

1	**9**	2	**28%**	19%	**1**	0	Fibromatous

2	**8,5**	3	**23%**	20%	**1**	0	Fissured

3	**6**	3	**31%**	22%	**2**	0	Gravidic

4	**10**	4	**19%**	9%	**1**	0	Peripheral ossification

5	**11**	2,5	**25%**	14%	**2**	0	Gigant-cell

6	**7**	2,5	**21%**	18%	**1**	0	Peripheral ossification

7	**12**	3	**27%**	25%	**1**	0	Fissured

8	**10**	4	**40%**	33%	**3**	1	Gigant-cell

9	**7**	2,5	**51%**	38%	**3**	1	Gravidic

10	**8**	3	**45%**	23%	**3**	1	Fibromatous

11	**6**	2	**47%**	27%	**3**	1	Fissured

12	**9**	3,5	**55%**	35%	**3**	1	Gigant-cell

13	**9**	3	**60%**	46%	**3**	0	Fissured

14	**10**	3	**73%**	45%	**3**	1	Gravidic

15	**12**	4	**65%**	31%	**3**	1	Fibromatous

16	**8**	4	**68%**	29%	**3**	1	Fibromatous

17	**12**	2,5	**78%**	34%	**3**	0	Fissured

18	**12**	3	**71%**	38%	**3**	1	Fibromatous

19	**10**	3	**62%**	29%	**3**	1	Peripheral ossification

20	**9**	3	**64%**	32%	**3**	1	Fibromatous

### Surgical treatment

After local anesthesia, intrasulcular incisions were made at the buccal and lingual sides with Bard-Parker surgical blade n° 15, at least one tooth away from the mesial portion, distally to the graft site, to create access for the tools and facilitate the direct clinical view of the defect.

A full-thickness flap was elevated and the granulation tissue was removed showing the true extension and depth of the periodontal defect.

On the palatal aspect, the size of the grafts was measured using a periodontal probe (XP 23/UNC15, Hu-Friedy MFG-Co, Inc., Chicago, IL, USA).

**Figure 2:**
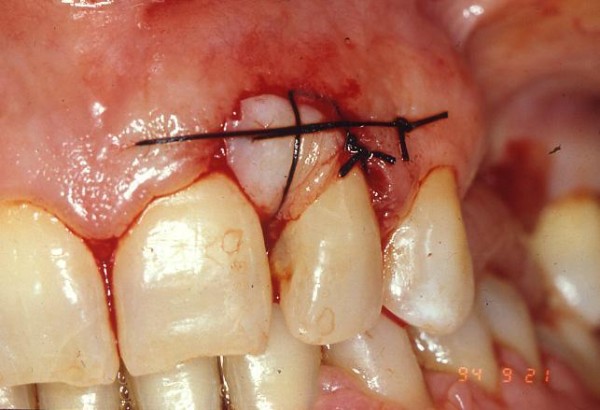
**The graft was positioned in the host site with stabilizing periosteal silk suture**.

The autograft, obtained from the donor site (in our case, the palatine mucosa of the maxillary pre-molars) and free from keratinized tissue, were positioned in the host site consisting of bone and periosteum (fig. 2)

**Figure 3:**
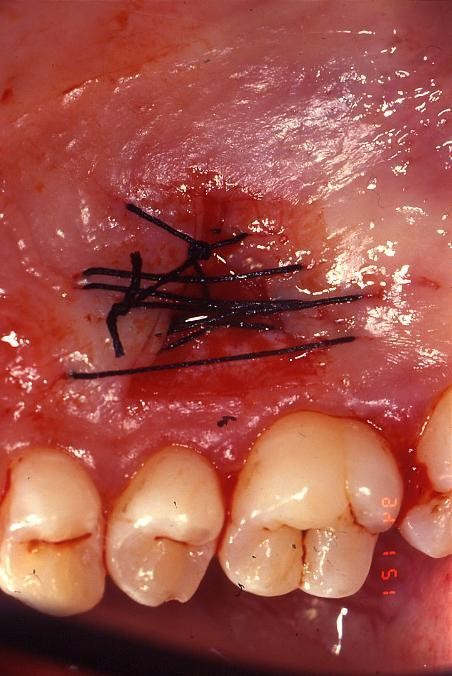
**Palatal view of the donor site**.

The graft were preserved in sterile physiological solution and then cleaned from the adipose tissue; it was stabilized with stabilizing periosteal silk suture (fig. 2; 3).

Finally, the flap were re-positioned and sutured with single stitches; in our protocol, the donor area was closed by three interrupted sutures in 3-0 silk (two at the borders and one in the centre) before grafting the recipient site.

Firm pressure were exerted with fingers for 2 - 3 minutes using a gauze dipped in physiological solution, to reduce the blood clot and promote healing.

All patients were placed on the following medication: azithromycin 500 mg once a day, for 3 days.

Sutures were removed from the donor site after 1 week.

During sutures removal, no important tissues inflammations were observed.

All bioptical samples were analyzed by the pathologist.

### Maintenance and follow-up

After surgical procedures, patients were instructed to rinse their mouths twice daily with 10 ml of and 0,12% chlorexidine (Corsodyl mothwash^® ^- GlaxoSmithKline -Brantford, Middlesex, UK) rinse for 1 min, 3 times a day, for 6 weeks.

Discomfort was assessed as the level of pain experienced by the patients during the postoperative first week due to the palatal wound by Visual analogue scale (**VAS**).

Three-point VAS ('none', 'mild or moderate', 'severe') was used to record discomfort levels reported by the participants.

The first day from surgery a number of 13 patients referred for mild discomfort and 7 for a severe discomfort.

At five days from surgery 15 patients referred for none discomfort and 5 for mild discomfort.

At a 10-day follow-up, post-operative clinical assessment demonstrated a G.I. grade of 0 or 1.

The participants in the study did not receive any dietary guidance except for the day of the surgery itself, when a diet based on soft and cold foods was suggested, taking care to chew on the opposite side of the mouth with respect to the donor site for the first week.

The patients then underwent a rigorous follow-up schedule at 30 days to assess the PI and GI, and to perform periodontal debridement. Complete epithelialisation of the palatal wound occurred in all patients only 4 weeks after surgery. At the 6 months follow-up visit the assessments of all the indexes were repeated. The PI and GI notably improved in most patients (Table 2).

**Figure 4:**
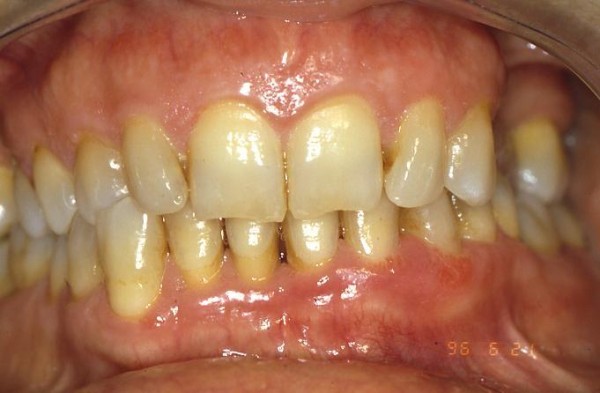
**Clinical aspect two years after surgery**.

**Figure 5:**
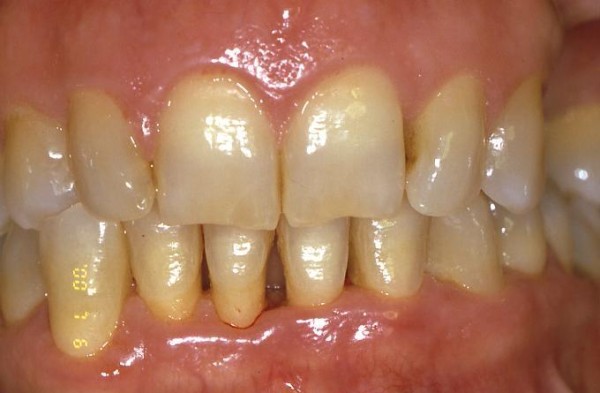
**Follow-up at six years later**.

**Figure 6:**
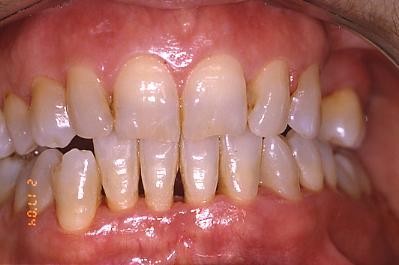
**Follow-up at 10 years: an aesthetically satisfactory gingival appearance and no signs of recurrence**.

The follow-up schedule involved visits at 1 year, 2,3,4,5,6,7,8,9 and10 years from the procedure (fig. 4; 5; 6).

## Results and Discussion

All patients referred that the post-operative course was free from any complication either at the donor or the host site.

After 10 years from the procedure, all patients had an aesthetically satisfactory gingival appearance and no sign of recurrence.

All the grafts were well vascularized and aesthetically satisfactory.

Unlike the classic approach, the surgical technique here described involved the use of a free gingival graft obtained from the palatine mucosa to cover the tissue gap in the host site.

The palatal mucosa in the premolar region is the ideal area for obtaining a graft for anatomic reasons [24], as an adequate thickness of the graft is ensured [25] without causing any damage to the greater palatine artery.

The main disadvantages of free soft tissue techniques are the double surgical wound and the relative discomfort suffered by the patient.

An other Author proposed the trap-door technique with the aim of keeping the epithelial layer intact to achieve healing by primary intention in the donor area [26].

This method was described as more conservative and less traumatic for the patient with localized GO, ensuring healing by primary intention and reducing palatal discomfort as reported in VAS table (Table 3).

**Table 3:** Visual analogue scale (VAS) table.

PATIENTS	D1	D5
1	Mild	None

2	Mild	None

3	Mild	None

4	Severe	Mild

5	Mild	None

6	Severe	Mild

7	Mild	None

8	Mild	None

9	Mild	None

10	Mild	None

11	Severe	Mild

12	Severe	Mild

13	Mild	None

14	Severe	None

15	Severe	None

16	Mild	None

17	Severe	Mild

18	Mild	None

19	Mild	None

20	Mild	None

Three-point VAS ('none', 'mild or moderate', 'severe') were used to record discomfort (D) levels reported by the 20 patients at 1 day from surgery (D1) and at 5 days (D5) from surgery.

Not only did this markedly improve the patients comfort but it also yield an aesthetically satisfying result thanks to the width and thickness of the keratinized tissue used, as well as safeguarding the site from the risk of recurrence [5].

The muco-gingival complex showed no functional or aesthetic damage and no bone reabsorption occurred, for exposure of the root surfaces in the involved area [10].

In the twenty treated cases, not only did no recurrence develop, but no further surgical correction was required.

Although the role of plaque has not been clearly defined consistently with other several authors [17], the hyper plastic tissue tends to aid plaque accumulation and to inhibit plaque removal, increases the gingival inflammation.

A treatment protocol including careful training in oral hygiene, combined with a valid surgical technique is therefore essential to resolve the problem of localized gingival overgrowth.

The additional use of chlorhexidine both in the initial and the maintenance therapy to ensure clinical control of plaque was also highly beneficial.

All patients need to be instructed in the correct use of oral hygiene measures and above all, to undergo regular professional prophylactic treatment.

The role of the dental hygienist was fundamental for co-adjuvant support therapy, and in ensuring good patient compliance [17].

## Conclusions

There are many reasons for GO.

Mostly, proper oral hygiene is sufficient to achieve normal healthy gum..

In some situations, however, gingival hyperplasia is drug-induced or can be a manifestation of a genetic disorder. In the latter, it may exist as an isolated abnormality or as part of a syndrome.

In our study, We suggest an alternative surgical protocol that seems to yield good aesthetic results and a stable muco-gingival complex in localized GO.

This technique is not appropriate in generalized GO, for the discomfort due to the multiple surgical sites necessary for the procedure.

The patient overgrowth, generalized or localized, should always be considered when choosing a course of treatment.

Follow-up at 10 years demonstrated excellent gingival health, satisfactory aesthetic results and no recurrence of the lesions.

## Competing interests

The authors declare that they have no competing interests.

## Authors' contributions

AB, AS and FRG made substantial contributions to conception and design and drafted the manuscript. SC, LP and FP revised it critically for important intellectual content and gave final approval of the version to be published. VC, TR and FP help in the patients follow-up. AD and MVB documented this study with digital pictures. GMN and RGC assisted the clinical procedures and selected the cases reported. All authors read and approved the final manuscript.

## Consent Statement

Written informed consent was obtained from the patients for publication of this study and accompanying images. A copy of the written consent is available for review by the Editor-in-Chief of this Journal.
